# Overexpression of CHD1L is positively associated with metastasis of lung adenocarcinoma and predicts patients poor survival

**DOI:** 10.18632/oncotarget.5070

**Published:** 2015-08-24

**Authors:** Li-Ru He, Ning-Fang Ma, Jie-Wei Chen, Bin-Kui Li, Xin-Yuan Guan, Meng-Zhong Liu, Dan Xie

**Affiliations:** ^1^ The State Key Laboratory of Oncology in South China, Sun Yat-Sen University Cancer Center, Collaborative Innovation Center for Cancer Medicine, Guangzhou, China; ^2^ Department of Radiation Oncology, Sun Yat-Sen University Cancer Center, Guangzhou, China; ^3^ Department of Pathology, Sun Yat-Sen University Cancer Center, Guangzhou, China; ^4^ Department of Histology and Embryology, State Key Laboratory of Respiratory Disease, Guangzhou Medical University, Guangzhou, China; ^5^ Departments of Clinical Oncology, The University of Hong Kong, Hong Kong, China

**Keywords:** non-small-cell lung carcinoma, CHD1L protein, metastasis, prognosis, gene amplification

## Abstract

*CHD1L* (chromodomain helicase/ATPase DNA binding protein 1-like gene) has been demonstrated as an oncogene in hepatocellular carcinoma (HCC), however, the role of *CHD1L* in non-small-cell lung cancer (NSCLC) tumorigenesis hasn't been elucidated. In this study, the expression and amplification status of *CHD1L* were examined by immunohistochemistry and fluorescence in situ hybridization respectively in 248 surgically resected NSCLCs. The associations between CHD1L expression and clinicopathologic features and the prognostic value of CHD1L were analyzed. Overexpression and amplification of *CHD1L* was found in 42.1% and 17.7% of NSCLCs, respectively. The frequency of *CHD1L* overexpression (53.2% *vs*. 28.1%, *P* = 0.002) and amplification (25.2% *vs*. 8.2%, *P* = 0.020) in adenocarcinoma (ADC), was much higher than that in squamous cell carcinoma (SCC). CHD1L overexpression was associated closely with ascending pN status (*P* < 0.001), advanced clinical stage (*P* = 0.001) and tumor distant metastasis (*P* = 0.001) in ADCs, but not in SCCs. For the whole cohort and ADC patients, univariate survival analysis demonstrated a significant association of CHD1L overexpression with shortened survival; and in multivariate analysis, CHD1L overexpression was evaluated as a independent predictor for overall survival and distant metastasis free survival. These results suggested that overexpression of CHD1L is positively associated with tumor metastasis of lung ADC, and might serve as a novel prognostic biomarker and potential therapeutic target for lung ADC patients.

## INTRODUCTION

Lung cancer is the leading cause of cancer deaths worldwide [1]. Non-small-cell lung cancer (NSCLC), which accounts for almost 80% of such death, is a very heterogeneous group of malignancies [2]. Even for earlier stages patients, a significant proportion of them will suffer from local recurrence and/or distant metastasis after radical surgery [3]. However, the international staging system is still inadequate to reliably predict patients' prognosis. Since chromosomal aberrations are believed to play an important role in tumor progression [4], it will be of great value to search the specific gene alterations in NSCLC which can provide additional staging information to optimize individual therapy.

Chromodomain helicase/ATPase DNA binding protein 1-like gene (*CHD1L*) is a newly identified oncogene that we previously isolated from a frequently amplified region at chromosome 1q of human hepatocellular carcinoma (HCC) [5]. A series of our further studies demonstrated that *CHD1L* contributes to HCC cell migration, invasion and metastasis, and is positively associated with tumor progression in HCC patients [5–7]. Recently, CHD1L has also been reported as a novel biomarker for patients' prognosis in several types of solid tumor, including breast cancer [8], gastric cancer [9], colorectal cancer [10], bladder cancer [11] and ovarian cancer [12]. To date, however, the abnormalities of *CHD1L* and its oncogenic role in NSCLC have not been studied. Since amplification of 1q, in which the *CHD1L* gene is located, was frequently examined in NSCLC and correlated closely with tumor recurrence and metastasis [13, 14], we conducted this study to examine the expression and amplification dynamics of *CHD1L* in a large cohort of NSCLC patients, and its clinicopathologic and prognostic significance was further evaluated.

## RESULTS

### Expression of CHD1L in NSCLCs

**Figure 1:**
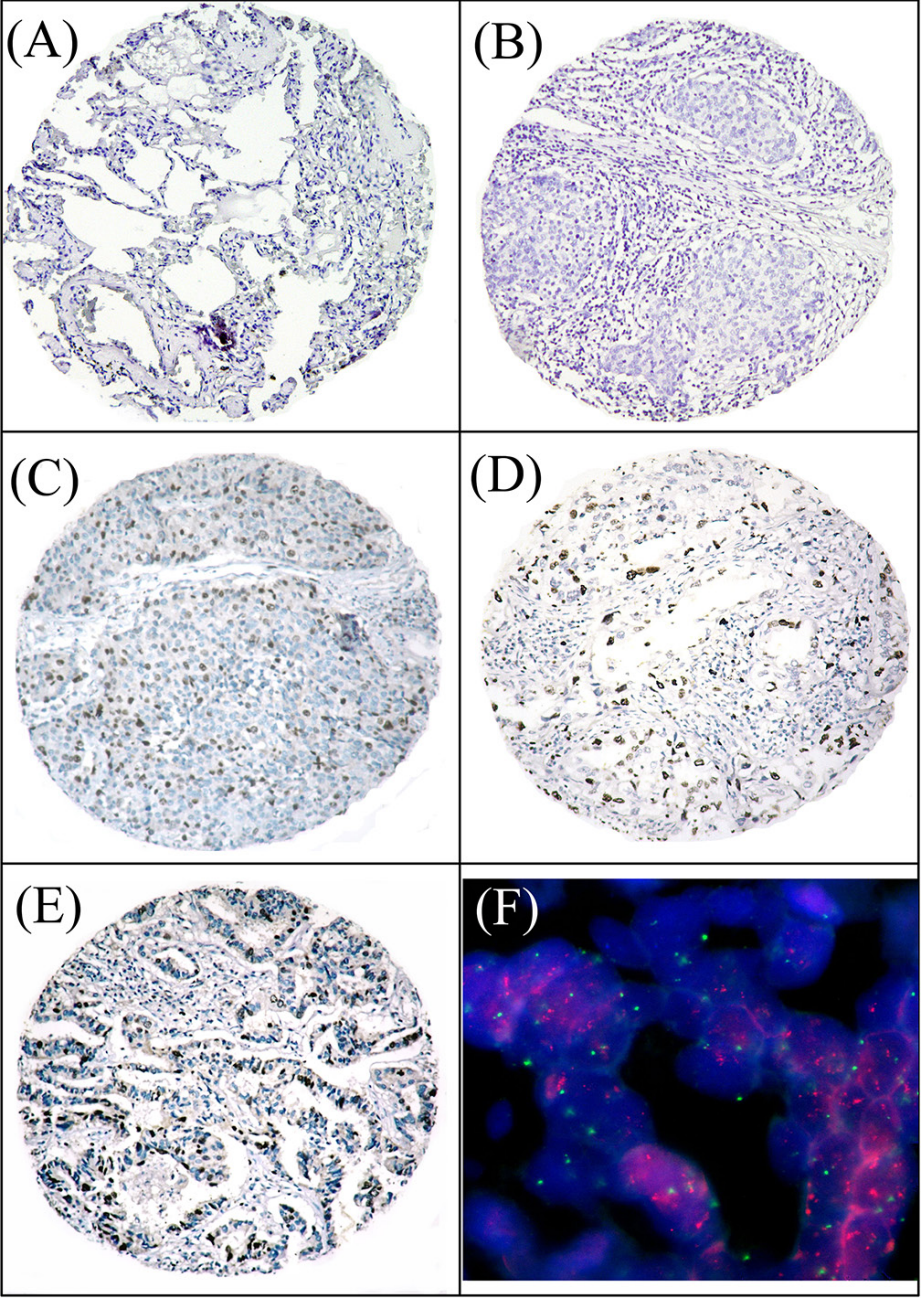
Immunohistochemical stainings of CHDIL and FISH assay of *CHDIL* gene in NSCLC tissues **A.** A normal lung tissue (case 19) showed a negative staining of CHDIL in all the epithelium cells (×100). **B.** A squamous carcinoma of lung (case 115) showed normal expression of CHDIL with a negative staining of CHDIL in all tumor cells (×100). **C.** Overexpression of CHDIL was detected in a squamous carcinoma of lung (case 216), in which about 50% of tumor cells were observed moderate positive staining of CHDIL (×100). **D.** Overexpression of CHDIL was detected in an adenocarcinoma of lung (case 57), in which about 40% of tumor cells were observed strong positive staining of CHDIL (×100). **E.** Overexpression of CHDIL was examined in an adenocarcinoma of lung (case 113), in which about 60% of tumor cells showed strong positive staining of CHDIL (×100). **F.** Amplification of *CHDIL* gene was examined by FISH in the same case of lung adenocarcinoma (case 113), in which *CHDIL* gene signals (red) were detected at least 3 times more than chromosome 1 centromere signals (green) (×1000).

The expression of CHD1L could be informatively examined by IHC in 233/248 (93.9%) of the NSCLCs and 27/30 (90.0%) of normal lung tissues. The non-informative samples included unrepresentative samples, samples with too few tumor cells (<300 cells per case) and lost samples, which we didn't use in our data compilation. Since the expression scores of CHD1L in normal lung tissues varied from 0 to 2 (intensity 0–2, proportion 0–1), overexpression of CHD1L was designated when the score was equal or more than 3. Using this criteria, CHD1L overexpression was observed in 98/233 (42.1%) of the NSCLCs, 58/109 (53.2%) of the adenocarcinomas (ADCs) and 25/89 (28.1%) of the squamous cell carcinomas (SCCs), respectively (Fig. 1).

### Association between CHD1L expression and clinic-pathological variables in NSCLCs

The associations between CHD1L expression and several clinico-pathological variables in NSCLC patients are assessed and displayed in Table 1. Overexpression of CHDIL in NSCLCs was significantly associated with tumor histology (*P* = 0.002), advanced pN status (*P* < 0.001) and advanced stage (*P* < 0.001). There was no significant association between CHD1L expression and other clinic-pathological features, such as patients' age, gender, tumor grade, pT status, surgical procedure, adjuvant chemotherapy and adjuvant radiotherapy. We further evaluated the associations in ADC and SCC patients, respectively, and found that the significant associations between CHD1L overexpression and advanced pN status/advanced stage were only seen in ADC patients, but not in SCC cases (Table 1).

**Table 1:** CHD1L expression and clinic-pathological variables

Characteristics	Total cases	Informative	CHD1L protein	
			Overexpression (%)	P*
Age (years)				0.780
≤59^†^	130	119	49 (41.2)	
>59	118	114	49 (43.0)	
Gender				0.190
Male	181	172	68 (39.5)	
Female	67	61	30 (49.2)	
Tumor grade				0.521
G1	24	22	8 (36.4)	
G2	173	164	67 (40.9)	
G3	51	47	23 (48.9)	
Histology				0.002
SCC	94	89	25 (28.1)	
ADC	113	109	58 (53.2)	
Others^‡^	41	35	15 (42.9)	
pT status				0.139
pT1–2	147	139	53 (38.1)	
pT3–4	101	94	45 (47.9)	
pN status				<0.001
pN0	120	113	31 (27.4)	
pN1	49	46	20 (43.5)	
pN2	79	74	47 (63.5)	
Stage				<0.001
I	82	80	22 (27.5)	
II	59	57	20 (35.1)	
III	107	96	56 58.3)	
Surgical procedure				0.405
Lobectomy	223	209	86 (41.1)	
Pneumonectomy	25	24	12 (50.0)	
Adjuvant chemotherapy				0.126
Yes	89	82	40 (48.8)	
No	159	151	58 (38.4)	
Adjuvant radiotherapy				0.670
Yes	35	33	15 (45.5)	
No	213	200	83 (41.5)	
SCC subgroup				
pN status				0.753
pN0	49	48	12 (25.0)	
pN1	18	17	5 (29.4)	
pN2	27	24	8 (33.3)	
Stage				0.568
I	33	32	8 (25.0)	
II	25	22	5 (22.7)	
III	36	35	12 (34.3)	
ADC subgroup				
pN status				<0.001
pN0	53	49	15 (30.6)	
pN1	22	22	12 (54.5)	
pN2	38	38	31 (81.6)	
Stage				0.001
I	38	37	12 (32.4)	
II	27	25	12 (48.0)	
III	48	47	34 (72.3)	

* Chi-square test

† Mean age

‡ Others include adenosquamous cell carcinoma, anaplastic large-cell carcinoma, sarcoma, adenoid cystic carcinoma, mucoepidermoid carcinoma and carcinoid tumor.

Abbreviation: CHD1L, chromodomain helicase/ATPase DNA binding protein 1-like gene; G, grade; SCC, squamous cell carcinoma; ADC, adenocarcinoma; pT, pathological tumor; pN, pathological node.

### Association between CHD1L expression and post-surgical failure patterns

During the median observation period of 46.1 months (range 3.6–199.3 months) for all patients, 67 experienced local-regional failure, 109 experienced distant metastasis, and 173 had cancer-specific death. The failure patterns of the NSCLC patients are presented in Table 2. The incidence of distant metastasis was much higher in patients with CHD1L overexpression than those with CHD1L normal expression for all patients (56.9% vs. 43.1%, *P* < 0.001); however, in subgroup analysis, this significant association was only seen in ADC patients (*P* < 0.001) and not in SCC patients (*P* = 0.162). No significant incidence difference was found in local-regional recurrence between groups with CHD1L normal/overexpression for all patients, SCC patients or ADC patients (*P* > 0.05, Table 2).

**Table 2:** Comparison of the failure patterns between NSCLC patients with CHD1L overexpression and normal expression

		No.	CHD1L protein		
			Normal expression (%)	Overexpression (%)	P*
All patients					
Local-regional recurrence	No	166	102 (61.4)	64 (38.6)	0.088
	Yes	67	33 (49.3)	34 (50.7)	
Distant metastasis	No	124	88 (71.0)	36 (29.0)	<0.001
	Yes	109	47 (43.1)	62 (56.9)	
SCC					
Local-regional recurrence	No	58	45 (77.6)	13 (22.4)	0.103
	Yes	31	19 (61.3)	12 (38.7)	
Distant metastasis	No	63	48 (76.2)	15 (23.8)	0.162
	Yes	26	16 (61.5)	10 (38.5)	
ADC					
Local-regional recurrence	No	81	40 (49.4)	41 (50.6)	0.356
	Yes	28	11 (39.3)	17 (60.7)	
Distant metastasis	No	48	31 (64.6)	17 (35.4)	0.001
	Yes	61	20 (32.8)	41 (67.2)	

* Chi-square test.

Abbreviation: CHD1L, chromodomain helicase/ATPase DNA binding protein 1-like gene; NSCLC, non-small-cell lung carcinoma; SCC, squamous cell carcinoma; ADC, adenocarcinoma.

### The impact of CHD1L expression on NSCLC patients' survival

**Figure 2:**
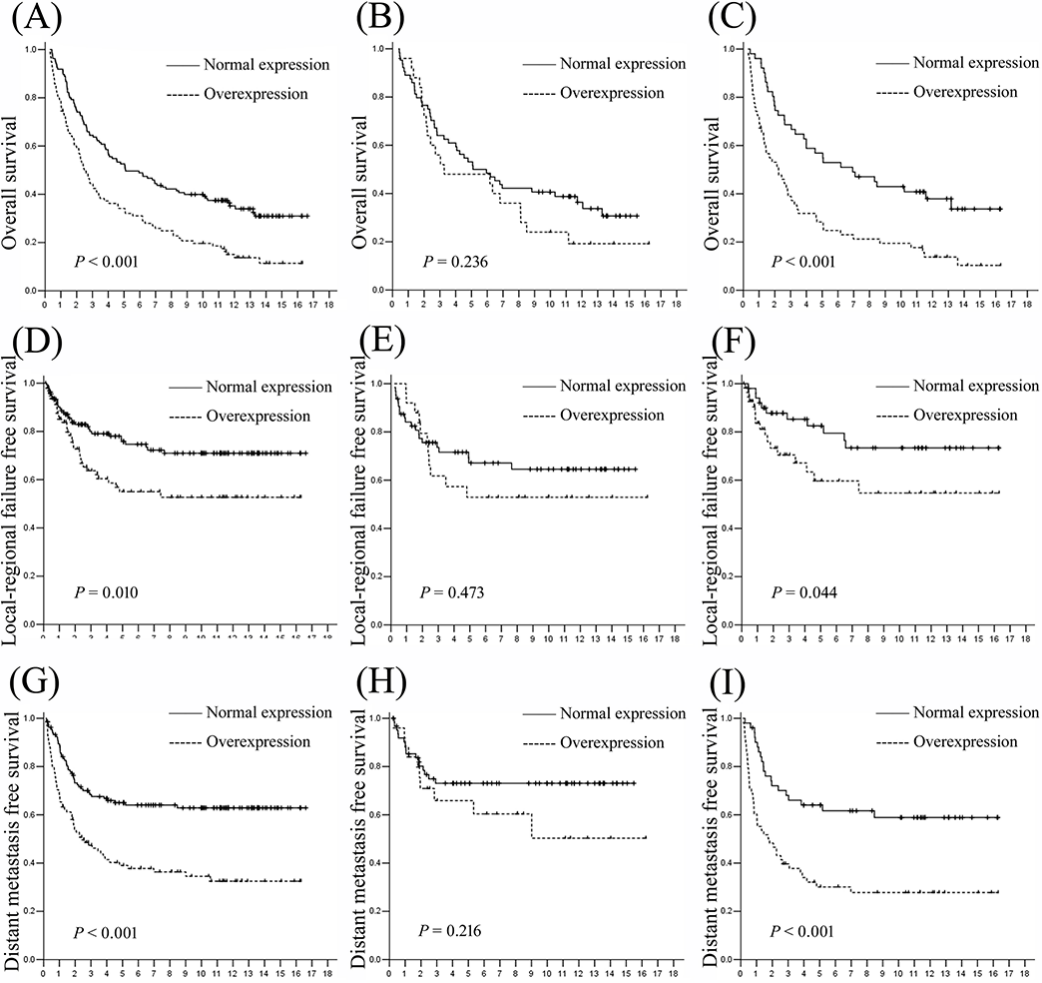
Survival curves according to CHDIL expression level for NSCLC patients **A, B.** and **C.** overall survival for the whole cohort, SCC and ADC patients; **D, E.** and **F.** local-regional failure free survival for the whole cohort, SCC and ADC patients; and **G, H.** and **I.** distant metastasis free survival for the whole cohort, SCC and ADC patients. CHDIL normal expression groups are shown as solid lines; CHDIL overexpression groups are shown as dashed lines.

In univariate analysis, CHD1L overexpression was evaluated to correlate closely with shorten overall survival (OS), shorten local-regional failure free survival (LRFFS) and shorten distant metastasis free survival (DMFS) for the whole cohort and the ADC patients, but not for the SCC patients (Fig. 2). Besides CHDIL overexpression, the impact value of age, gender, tumor grade, histology, stage, surgical procedure, adjuvant chemotherapy and adjuvant radiotherapy have also been tested in univariate analysis for OS, LRFFS and DMFS, respectively. Variables that showed a significant impact on patients' survival for the whole cohort and the ADC patients in univariate analysis were listed in Table 3, which were further tested in multivariate analysis. Other variables, including tumor grade, surgical procedure, adjuvant chemotherapy and adjuvant radiotherapy, were not evaluated as significant prognostic factors in univariate analysis (Data not shown). Our multivariate analysis results showed that CHD1L overexpression was evaluated as an independent predictor of poor OS and poor DMFS for the whole cohort and ADC patients (Table 3).

**Table 3:** Multivariate Cox regression analysis for OS, LRFFS and DRFS

Variables	OS		LRFFS		DMFS	
	HR (95%CI)	*P*	HR (95%CI)	*P*	HR (95%CI)	*P*
ALL patients						
Age^†^	1.406 (1.042–1.897)	0.026	–	–	1.379 (0.945–2.011)	0.095
Histology^‡^	1.269 (0.924–1.744)	0.142	–	–	2.154 (1.375–3.372)	0.001
Stage^§^	1.745 (1.436–2.121)	<0.001	3.365(2.284–4.958)	<0.001	1.764 (1.374–2.264)	<0.001
CHD1L expression^¶^	1.526 (1.120–2.079)	0.007	1.432(0.881–2.328 )	0.148	1.755(1.185–2.599)	0.005
ADE patients	–	–	–	–	–	–
Age^†^	1.295 (0.836–2.005)	0.247	–	–	–	–
Stage^§^	1.994 (1.477–2.691)	<0.001	5.800(2.779–12.107)	<0.001	2.102 (1.480–2.984)	<0.001
CHD1L expression^¶^	1.720 (1.091–2.712)	0.020	1.478(0.681–3.208)	0.323	2.019 (1.168–3.491)	0.012

† Age > 59 yrs *vs*. Age ≤ 59 yrs

‡ Non squamous cell carcinoma *vs* Squamous cell carcinoma

§ Stage III *vs.* Stage II *vs.* Stage I

¶ Overexpression *vs.* Normal expression.

Abbreviation: CHD1L, chromodomain helicase/ATPase DNA binding protein 1-like gene; OS, overall survival; LRFFS, local-regional failure free survival; DMFS, distant metastasis free survival.

### Amplification of *CHD1L* in NSCLCs

The FISH analysis was informative in 50.0% (15/30) of the normal lung tissues and 52.4% (130/248) of the NCSLCs. The main reasons for most of the non-informative cases were samples without FISH signal and samples with weak target signals or those with a strong signal background. Amplification of *CHD1L* was not detected in any of the normal lung tissues, but was observed in 17.7% (23/130) of the informative NSCLCs. In univariate analysis by Cox regression models, *CHD1L* amplification was evaluated as a significant predictor for distant metastasis (hazard ratios (HR) = 3.255, 95% confidence intervals (CI) = 1.984–5.339, *P* < 0.001), recurrence (HR = 2.795, 95% CI = 1.426–5.481, *P* = 0.003) and OS (HR = 3.185, 95% CI = 2.039–4.975, *P* < 0.001).

In our study, 117 cases were shown to be simultaneously informative by both IHC and FISH tests. Amplification of *CHD1L* gene was significantly associated with CHD1L protein overexpression (*P* < 0.001) and tumor histology (*P* = 0.020, Table 4). The frequency of *CHD1L* amplification in ADC (25.5%) was much higher than that in SCC (8.2%). Overexpression of CHD1L was observed in the majority (16/19, 84.2%) of NSCLCs with *CHD1L* amplification; whereas, CHD1L overexpression can also be detected in 35.7% (35/98) of the NSCLCs without *CHD1L* gene amplification.

**Table 4:** Association of *CHD1L* expression and amplification in NSCLC

	Informative cases	*CHD1L* gene		P*
		No amplification	Amplification	
CHD1L protein				<0.001
Normal expression	66	63 (95.5)	3 (4.5)	
Overexpression	51	35 (68.6)	16 (31.4)	
Histology				0.020
SCC	49	45 (91.8)	4 (8.2)	
ADC	55	41 (74.5)	14 (25.5)	

* Chi-square test.

Abbreviation: CHD1L, chromodomain helicase/ATPase DNA binding protein 1-like gene; NSCLC, non-small-cell lung carcinoma; SCC, squamous cell carcinoma; ADC, adenocarcinoma.

## DISCUSSION

In this study, our results showed that the expression of CHDIL in all of the normal lung tissue specimens was absent or at low levels; in contrast, overexpression of CHDIL was frequently examined in many of the NSCLC specimens. Moreover, the frequency of CHDIL overexpression increased with an ascending pN status and advancing clinical stage in NSCLC, especially in lung ADCs. Similar results were also observed in other types of human cancer [6, 8, 9]. In HCC, overexpression of CHD1L was associated closely with microsatellite formation and venous invasion [6]. In gastric cancer, up-regulated expression of CHD1L was reported to be correlated with tumor depth, nodal involvement and distant metastasis [9]. While in breast cancer, presence of CHD1L expression was associated with higher Ki-67 index and HER2 amplification [8]. These findings suggest that increased expression of CHD1L is associated with aggressive tumor biology in certain types of human solid tumors, including lung ADC.

Interestingly, we found that the expression level of CHDIL in NSCLCs was significantly associated with tumor histology. On the basis of cell morphology, ADC and SCC are the two most common types of NSCLC. Given the difference in biological and clinical behavior of the two types of NSCLC, the patterns of chromosomal aberrations in ADC and SCC are thought to be different to some extent [15]. One of the differences between the 2 types of NSCLC is that 1q amplification was more common in ADC as compared to that in SCC [14, 16, 17]. Since *CHD1L* is an oncogene locates at a frequently amplified region of 1q [5], our results which showed that the frequency of CHDIL overexpression and the frequency of *CHDIL* amplification in ADC were much higher than those in SCC, is in agreement with the previous reports.

Most importantly, overexpression of CHD1L was significantly associated the present of post-operation distant metastasis, and was evaluated as an independent adverse predictor of OS and DMFS for lung ADC patients. As we know, lung ADC is a highly malignant disease which tends to disseminate haematogeneously at an early stage. Even after a curative resection, the majority patients still die within 5 years because of tumor metastasis [1], thus, effective adjuvant treatment are actually needed. However, the benefit of adjuvant chemotherapy is still limited [18, 19], and the available target therapy agents are quite rare. Thus, identification of more potential therapeutic targets is still highly desirable. When compared the chromosomal imbalances between primary and metastatic lung ADC, Goeze et al [14] reported that DNA overrepresentation on 1q was the most common alteration. And the overrepresentations on 1q21–q25 were evaluated to be associated with the metastatic phenotype of lung ADC [14]. Our results, together with these findings, suggest that up-regulated expression of *CHD1L*, a gene locates at 1q21, may provide a selective advantage in metastasis process of lung ADC; and *CHD1L* might be served as a potential therapeutic target for lung ADC patients.

With regard to the mechanism of up-regulated protein expression of CHD1L in NSCLCs, it is well established that overexpression of an oncogene is often caused by DNA amplification. Amplification of *CHDIL* has been detected in 50.6% of HCCs [5] and 18.3% of colorectal cancer samples [10]. However, none of the previous studies has evaluated the association of *CHDIL* gene amplification and CHDIL protein overexpression in any types of human cancers. To determine whether or not overexpression of CHD1L in NSCLCs was caused by gene amplification, we further examined the amplification status of *CHD1L* by FISH and tested its association with CHD1L expression. We observed that overexpression of CHDIL protein was associated, but not always coincided with *CHDIL* gene amplification in NSCLC. This result indicates that gene amplification might be one of the mechanisms that produce an excess of CHDIL protein; however, the modification of CHDIL expression could also be regulated by other mechanisms, such as post-translational modification.

When come to the gene function of *CHD1L*, it has not been well clarified in human cancers except for HCC. Our previous studies showed that increased expression of CHD1L promoted tumor cell migration and metastasis by increasing cell motility and inducing epithelial-mesenchymal transition (EMT) in HCC [6]. And this procedure was completed via ARHGEF9-mediated Cdc42 activation [6]. More recently, we have also found that CHD1L promoted HCC cell invasiveness and metastasis by activating kazal-like domains proteoglycan 1 (SPOCK1) -AKt signaling pathway [20]. Collectively, both CHD1L-ARHGEF9-Cdc42-EMT pathway and CHD1L-SPOCK1-AKt pathway might be involved in HCC progression and metastasis. However, the specific underling mechanisms of *CHD1L* in lung ADC metastasis have not been investigated. Clearly, further functional studies are needed.

Our study did have some limitations. It is a retrospective study, we didn't include all the clinical information because of the unavailability of some data. And there was a high heterogeneity in the regimens and cycles of adjuvant chemotherapy that we could not further analyzed. Thus, further studies are needed to confirm these results.

In summary, our results provide some evidences for the concept that (1) overexpression of CHD1L might provide a selective advantage for distant metastasis of lung ADC; and (2) CHD1L plays an adverse role in the prognosis of lung ADC, and might serve as a novel prognostic marker and potential therapeutic target for lung ADC patients.

## PATIENTS AND METHODS

### Patients and tissue specimens

In this study, paraffin-embedded tissue samples from 248 NSCLC patients were obtained from the Pathology Department of Cancer Center, Sun Yat-sen University, Guangzhou, China, between February 1994 and January 1998. All the selected patients had received complete resection (223 had lobectomy and 25 underwent pneumonectomy) and with adequate follow-up data. Patients who had preoperative treatment or those who had a second malignant disease were excluded.

Data regarding stage was according to the pathology Tumor-Node-Metastasis (pTNM) system (AJCC/UICC 2007). Tumor differentiation and histotype were according to the World Health Organization classification for NSCLC. Local/regional failure was defined as the recurrence of the primary tumor and regional lymph nodes, while distant failure was defined as the metastasis to any site beyond the primary tumor and regional lymph nodes. The study was approved by the medical ethics committee of Cancer Center, Sun Yat-Sen University and was performed in accordance with the Declaration of Helsinki.

### Construction of tissue microarrays (TMA)

The TMA was constructed according to a method described previously [21]. Briefly, the formalin-fixed, paraffin-embedded tissue blocks and the corresponding histological H&E stained slides were overlaid for tissue TMA sampling. The tissues (248 NSCLC and 30 normal lung tissues taken from regions that were not affected of the same patients) were sampled using a tissue arraying instrument (Beecher Instruments, Silver Spring, MD); a 0.6-mm-diameter cylinder of tissue was removed. Subsequently, we re-embedded the tissue cylinder into a predetermined position in a recipient paraffin block. Three cores of sample were selected from each primary NSCLC and normal lung tissue, and multiple sections (5 μm thick) were cut from the TMA block and mounted on microscope slides.

### Immunohistochemistry (IHC)

The IHC study of CHD1L was performed using a standard streptavidinperoxidase method as previously described [12]. The TMA sections were deparaffinized and rehydrated. The endogenous peroxidase activity was blocked with 3% H2O2 for 10 minutes. For the antigen retrieval, slides were immersed in 10 mM citrate buffer (pH 6.0) and boiled in a microwave oven for 15 minutes. Non-specific binding was blocked by 5% normal goat serum for 10 minutes. The slides were incubated with a 1:100 dilution of monoclonal antibody against CHD1L (Abcam) at 4°C overnight in a moist chamber. The slides were sequentially incubated with biotinylated goat anti-mouse IgG (1:100 dilution; Santa Cruz Biotechnology) and then streptavidin-peroxidase conjugate, each for 30 minutes at room temperature. Isotope-matched human IgG was used in each case as a negative control. Finally, the 3, 5-diaminobenzidine (DAB) Substrate Kit (Dako) was used for color development followed by Mayer hematoxylin counterstaining.

Positive expression of CHD1L in normal and malignant lung tissues was primarily a nuclear pattern (Fig. 1). For the evaluation of CHD1L staining, a semi-quantitative scoring criterion was used, in which both staining intensity and positive cells percentage were recorded [9, 12]. A staining index (with values from 0 to 12) was obtained as the intensity of CHDIL staining (0 = negative, 1 = weakly positive, 2 = moderate positive, 3 = strongly positive) times the proportion of immunopositive tumor cells (0% = 0, <10% = 1, 10% to <50% = 2, 50% to <75% = 3, ≥75% = 4). A minimum of 300 epithelial cells was counted for each case. Two independent pathologists (Dr. Xie D and Chen JW) blinded to the clinicopathologic information performed the scorings. The inter-observer disagreements (about 6% of the total informative cases) were reviewed a second time, followed by a conclusive judgment by both pathologists.

### Fluorescence *in situ* hybridization (FISH)

Two-color FISH was applied to the sections of TMA. The BAC clone (RP11–337C18) at 1q21 containing the *CHD1L* gene probe and the chromosome 1 centromere probe were labeled with Spectrum-red and Spectrum-green (Vysis, Downers Grove, IL), respectively. The FISH reaction was performed as described previously with slight modification. Briefly, the deparaffinized TMA section was treated with proteinase K (400 μg/ml) at 37°C for 45 min, followed by denaturing in 70% formamide, 2 × SSC at 75°C for 6 min. Fifty nanograms of each probe were mixed in a 20μl hybridization mixture (containing 55% formamide, 2 × SSC, and 2 μg human Cot1 DNA), denatured at 75°C for 5 minutes and then hybridized to the denatured TMA section at 37°C for 24 hours. After washing, the TMA section was counterstained with 1 μg/ml DAPI in an anti-fade solution and examined with a Zeiss Axiophot microscope equipped with a triple-band pass filter. A minimum of 300 tumor cells was evaluated per specimen. Amplification of *CHD1L* gene were defined as the presence (in ≥20% of tumor cells) of either 6 (or more) gene signals or more than 3 times as many gene signals than reference chromosome 1 centromere signals (Fig. 1F) [22–24]. All samples not meeting the criteria for gain or amplification were considered normal. Control hybridizations to normal fibroblasts and to normal epithelial cells were performed to confirm that the hybridization efficiency of the test and reference probes was similar. The slides were assessed by the two independent pathologists (Dr. Xie D and Chen JW) mentioned above. The inter-observer disagreements (about 3% of the total informative cases) were reviewed a second time, followed by a conclusive judgment by both pathologists.

### Statistical analysis

Statistical analysis was performed with the SPSS software (SPSS Standard version 13.0, SPSS Inc. Chicago, IL). The association of CHD1L protein expression with NSCLC patient's clinicopathological features and its correlation with *CHD1L* gene amplification were assessed by the Chi-square test. DMFS, LRFFS and OS were defined as the time from the date of diagnosis to tumor metastasis, local-regional tumor recurrence and death, respectively. Survival curves were assessed by the Kaplan-Meier method and compared by the log-rank test. Cox regression analysis was carried out to assess the significance of variables for survival. Two-sided, *P* < 0.05 was considered statistically significant.
